# Ruminative reflection is associated with anticorrelations between the orbitofrontal cortex and the default mode network in depression: implications for repetitive transcranial magnetic stimulation

**DOI:** 10.1007/s11682-021-00596-4

**Published:** 2021-12-03

**Authors:** Tobin J. Ehrlich, Jyoti Bhat, Andrea M. Horwege, Daniel H. Mathalon, Gary H. Glover, Brian J. Roach, Bashar W. Badran, Steven D. Forman, Mark S. George, J. Cobb Scott, Michael E. Thase, Jerome A. Yesavage, Deborah A. Yurgelun-Todd, Allyson C. Rosen

**Affiliations:** 1 Veterans Affairs Palo Alto Health Care System, 3801 Miranda Ave (151Y), Palo Alto, CA 94304 USA; 2 University of Michigan, Ann Arbor, MI USA; 3 Palo Alto Veterans Institute for Research, Palo Alto, CA 94304 USA; 4 Mental Health Service, San Francisco Veterans Affairs Health Care System, University of California, San Francisco, San Francisco, CA USA; 5 Department of Psychiatry, University of California, San Francisco, San Francisco, CA USA; 6 Department of Radiology, Stanford University, Stanford, CA USA; 7 Northern California Institute for Research and Education, San Francisco Veterans Affairs Medical Center, University of California, San Francisco, San Francisco, CA USA; 8 Brain Stimulation Division, Department of Psychiatry, Medical University of South Carolina, Charleston, SC USA; 9 Department of Veterans Affairs, Veterans Affairs Medical Center, Pittsburgh, PA USA; 10 Western Psychiatric Institute and Clinic, University of Pittsburgh School of Medicine, Pittsburgh, PA USA; 11 Ralph H. Johnson VA Medical Center, Charleston, SC USA; 12 VISN4 Mental Illness Research, Education, and Clinical Center at the Corporal Michael J. Crescenz VA Medical Center, Philadelphia, PA 19104 USA; 13 Department of Psychiatry, Perelman School of Medicine, University of Pennsylvania, Philadelphia, PA USA; 14 Department of Psychiatry and Behavioral Sciences, Stanford University School of Medicine, Stanford, CA USA; 15 Rocky Mountain Network Mental Illness Research Education and Clinical Centers (VISN 19), VA Salt Lake City Health Care System, Salt Lake City, UT USA; 16 Department of Psychiatry, University of Utah School of Medicine, Salt Lake City, UT USA

**Keywords:** Rumination, Reflection, Treatment-resistant depression, Default mode network, Repetitive transcranial magnetic stimulation

## Abstract

Patients with depression who ruminate repeatedly focus on depressive thoughts; however, there are two cognitive subtypes of rumination, reflection and brooding, each associated with different prognoses. Reflection involves problem-solving and is associated with positive outcomes, whereas brooding involves passive, negative, comparison with other people and is associated with poor outcomes. Rumination has also been related to atypical functional hyperconnectivity between the default mode network and subgenual prefrontal cortex. Repetitive pulse transcranial magnetic stimulation of the prefrontal cortex has been shown to alter functional connectivity, suggesting that the abnormal connectivity associated with rumination could potentially be altered. This study examined potential repetitive pulse transcranial magnetic stimulation prefrontal cortical targets that could modulate one or both of these rumination subtypes. Forty-three patients who took part in a trial of repetitive pulse transcranial magnetic stimulation completed the Rumination Response Scale questionnaire and resting-state functional magnetic resonance imaging. Seed to voxel functional connectivity analyses identified an anticorrelation between the left lateral orbitofrontal cortex (−44, 26, −8; *k* = 172) with the default mode network-subgenual region in relation to higher levels of reflection. Parallel analyses were not significant for brooding or the RRS total score. These findings extend previous studies of rumination and identify a potential mechanistic model for symptom-based neuromodulation of rumination.

**Supplementary Information:**

The online version contains supplementary material available at 10.1007/s11682-021-00596-4.

## Introduction

Rumination, a disabling repetitive focus on symptoms of depression, has been related to abnormally high functional connectivity (FC) between the subgenual prefrontal cortex and the default mode network (DMN; Hamilton et al., 2015). There is an evolving literature indicating that repetitive pulse transcranial magnetic stimulation (rTMS) can alter FC in major depressive disorder (MDD; for a review see Beynel et al., 2020; Fox et al., 2014; Philip et al., 2018). Many early models of rTMS depression therapy hypothesize that the dorsolateral prefrontal cortex is dysfunctional in exerting cognitive control over other regions that mediate emotions (e.g., Lantrip et al., 2017). Whereas rTMS of the frontal lobe is an FDA approved approach to treat MDD, the most effective frontal targets within that region for different patients are not established. A number of studies demonstrate that frontal regions with the strongest negative correlation (anticorrelation) with the subgenual cingulate are relatively more effective targets in reducing depression (Cash et al., 2017; Fox et al., 2012; Mir-Moghtadaei et al., 2015; Weigand et al., 2018; Williams et al., 2018). These finding are consistent with brain activity in depressed patients, including abnormally high activity in the subgenual region (Berlim et al., 2014; Drobisz & Damborska, 2019; Mayberg et al., 1999; Morishita et al., 2014) and low activity in the frontal lobe (Baxter et al., 1989; George & Wassermann, 1994; Martinot et al., 2010). Thus, targeting regions with high anticorrelation between these regions may normalize this relationship. Identifying which frontal regions are functionally connected with the DMN-subgenual complex of regions (DMN-subgenual), and which are also associated with severity of rumination, could identify cortical regions that could potentially modulate this elevated connectivity and alter rumination. One challenge however, is that rumination is comprised of two distinct cognitive subtypes—reflection and brooding—that are both associated with worse current depression severity but different longitudinal outcomes (Nolen-Hoeksema & Morrow, 1991; Treynor et al., 2003). Reflection is an active problem-solving thought process that predicts later decreases in depression severity. Whereas increasing a form of rumination to treat depression may seem counterintuitive, reflection is believed to be compensatory to support recovery. In contrast, brooding is a passive comparison with an unachievable standard that predicts later increases in depression severity (Treynor et al., 2003). Knowing which of these subtypes of rumination are associated abnormal DMN-subgenual FC is thus important for directing clinical neuromodulation. In sum, if elevated DMN-subgenual FC plays a mechanistic role in rumination then identifying which rumination subprocess is related and which cortical target is connected will be important for modulating this system.

We aimed to perform a resting state fMRI analysis of data from a cohort of patients with treatment-resistant depression undergoing rTMS therapy (Yesavage et al., 2018) and identify a prefrontal cortical region in which the functional connection to the brain areas related to rumination (i.e., DMN-subgenual) is related to the self-reported level of rumination (i.e., reflection and/or brooding). Since reducing depression involves targeting a frontal region anticorrelated with a subgenual region, we hypothesized that a similar pattern of FC would be related to a process like reflection that is associated with reduced depression (Treynor et al., 2003). Specifically, the strength of self-reported reflection should be correlated with the strength of the frontal anticorrelation with the DMN-subgenual region. Conversely, since brooding is associated with increased depression (Treynor et al., 2003), the strength of self-reported brooding should be correlated with the strength of the frontal positive correlation with the DMN-subgenual region. To test these hypotheses, we defined a region of interest (ROI) based on a meta-analysis of rumination studies of MDD (Hamilton et al., 2015). This region of interest was comprised of two core regions of the DMN (posterior cingulate cortex and medial prefrontal cortex; Fox et al., 2005) and a subgenual region (Hamilton et al., 2015). We then used a seed to voxel analysis (Whitfield-Gabrieli & Nieto-Castanon, 2012) testing whether there was a cortical region, likely in the prefrontal cortex, whose correlation with this ROI was related to level of self-reported reflection and/or brooding. This cortical region could serve as a potential candidate region to target for modulation (Fox et al., 2014) and further supported the model of rumination as involving an interaction between a frontal lobe subregion and the DMN-subgenual region.

## Methods

### Participants

Forty-three Veterans with treatment-resistant depression (33 male; mean age = 54.98 [SD = 12.27] years, mean education = 13.9 [SD = 1.96] years; see Table 1 for demographic data), were enrolled in this sub-study as part of a larger prospective, randomized, sham-controlled rTMS for depression clinical trial (ClinicalTrials.gov ID: NCT01191333). A detailed description of the full inclusion and exclusion criteria for the trial was previously published (Mi et al., 2017; Yesavage et al., 2018). As part of the clinical trial, the clinician administered Hamilton Depression Rating Scale (Hamilton, 1960) and Clinician-Administered PTSD Scale for the DSM-IV (Blake et al., 1995) were completed to confirm the presence of major depressive disorder and to identify posttraumatic stress disorder (28% of the participants in this study met criteria for PTSD, see Table 1). These measures were sometimes completed at a remote time from MRI data acquisition, so that to enable evaluation of changes in symptom severity, brief self-report measures of depression (BDI-II) and posttraumatic stress disorder (PCL-M) were completed close in time to the MRI data acquisition, see questionnaires for additional detail. Participants were selected from patients who were willing and able to undergo fMRI data acquisition. All participants were screened for known neurological, comorbid psychiatric, and vascular risk factors or any medication which might affect vascular reactivity or cognitive performance.

**Table 1:** Demographic and questionnaire descriptive statistics

Characteristics	Mean (SD)
Age (years)	54.98 (12.27)
Education (years)	13.9 (1.96)
RRS-Reflection	11.28 (3.35)
RRS-Brooding	12.4 (3.79)
BDI-II	23.3 (10.26)
PCL-M	42.67 (17.23)
fMRI Post-Active Treatment (months)	21.07 (9.34)
	N (percentage)
Sex	
Male	33 (76.7%)
Female	10 (23.3%)
Handedness	
Right	38 (88.4%)
Left	5 (11.6%)
fMRI Post-Active Treatment	7 (16%)
PTSD Diagnosis	12 (28%)

*RRS* Rumination Response Scale, reflection and brooding subscales, *BDI-II* Beck Depression Inventory-II, *PCL-M* Posttraumatic Stress Disorder Checklist, Military Version, *fMRI Post-Active Treatment* subjects with fMRI scan acquired after active repetitive pulse transcranial magnetic stimulation treatment; PTSD Diagnosis is determined from the Clinician-Administered PTSD Scale

### Questionnaires

Participants completed the Rumination Response Scale (RRS; Nolen-Hoeksema & Morrow, 1991), Beck Depression Inventory-II (BDI-II; Beck et al., 1996), and the Posttraumatic Stress Disorder Checklist, Military Version (PCL-M; Weathers et al., 1993) within one week of fMRI data acquisition.

The RRS (Treynor et al., 2003) contains three subscales, two of which are unique to the cognitive subtypes of rumination- reflection and brooding. The third subscale contains depression related items and was not evaluated in this study since it was redundant with other more focused measures of depression (e.g., BDI-II). The RRS asks participants to indicate how often they think about or do mental and behavioral activities when they are depressed on a scale from low (1 = almost never) to high (4 = almost always) frequency. The reflection and brooding subscales include 5-items per subscale. The reflection subscale is thought to measure an attempt to decrease depression severity through problem-solving, with questions such as “Go away by yourself and think about why you feel this way.” The brooding subscale is thought to measure a passive comparison with others and unrealistic standards (Treynor et al., 2003). The brooding subscale includes items such as “Think about a recent situation and wishing it could have gone better.” The reflection subscale was shown to have a coefficient alpha of .72 and the brooding subscale was shown to have a coefficient alpha of .77 (Treynor et al., 2003). The RRS total score combines all three subscales into a composite score reflecting the construct of rumination. The RRS total score was shown to have a coefficient alpha of .90 (Treynor et al., 2003).

The BDI-II (Beck et al., 1996) is a 21-item, four-point, Likert-type scale that measures depression symptom severity over the past two weeks. Higher total scores are indicative of an increased number and severity of current depression symptoms. Scores from 0 to 13 indicate minimal depression, 14–19 indicates mild depression, 20–28 indicates moderate depression, and 29–63 indicates severe depression. The BDI-II has was shown to have a coefficient alpha of .92 (Beck et al., 1996).

The PCL-M (Weathers et al., 1993) is a 17 item, five-point (1 = not at all, 5 = extremely), Likert-type scale that measures posttraumatic stress disorder severity. The questions of the PCL-M are worded to assess military related trauma. Greater scores indicate a greater number and severity of posttraumatic stress disorder-related symptoms. The PCL-M was shown to have a coefficient alpha of .96 (Weathers et al., 1993).

### Data collection

Scanning was performed exclusively on 3 T MRIs across five imaging centers as follows: Siemens Verio (Pittsburgh, PA), Siemens Trio (Charleston, SC and Salt Lake City, UT), Siemens Skyra (San Francisco, CA), and a GE 3 T Discovery 750 scanner (Palo Alto, CA). All Siemens sites used a 12-channel head coil with the exception of San Francisco which used a 32-channel head coil, and the GE site used an 8-channel head coil. High-resolution structural MRI (T1) of approximately 1mm^3^ in-plane resolution was collected using the protocols from the Alzheimer’s Disease Neuroimaging Initiative (http://adni.loni.ucla.edu). The echoplanar fMRI pulse sequences were adapted from the functional biomedical informatics research network (fBIRN; Brown et al., 2011; Greve et al., 2011). Functional MRI scans were collected in an axial plane using an echoplanar sequence (TR 2 s, TE 30 ms, 77-degree flip angle, image resolution 3.44 × 3.44 × 3.5 mm, 30 4-mm slices) of eight minutes duration (240 repetitions). Seven participants completed six-minute runs (180 repetitions) prior to the decision to lengthen the sequence to improve data robustness. Before commencing data collection an fBIRN (Keator et al., 2016) phantom and a traveling subject were scanned and cross site image quality was evaluated by our lead MR physicist.

### fMRI preprocessing and FC analyses

Analyses were performed using the CONN Toolbox, version 18a (Chai et al., 2012; Whitfield-Gabrieli & Nieto-Castanon, 2012), which used Statistical Parametric Mapping, version 12 (http://www.fil.ion.ucl.ac.uk/spm/). Preprocessing of resting-state fMRI used the standard CONN pipeline, which included realignment and unwarping, centering to 0, 0, 0 coordinates, outlier detection, slice timing correction, segmentation and normalization into MNI space, and smoothing to 6-mm FWHM. Denoising to avoid spurious correlations caused by head motion and other signal changes unrelated to brain activity was completed. Problematic time points during the scan were removed using the Artifact Detection Tools (https://www.nitrc.org/projects/artifact_detect). Outlier images were removed if the head displacement in x, y, or z direction was greater than 0.9 mm from the previous frame, or if the global mean intensity in the image was greater than 5 standard deviations from the mean image intensity for the entire scan. Global signal regression, a widely used preprocessing method, was not used because it can produce negative correlations that can influence the presence of anticorrelations (Murphy et al., 2009) and contribute to spurious positive correlations (Saad et al., 2012). Additionally, with the use of the Artifact Detection Tools software the benefit that global signal regression offers for motion correction (Ciric et al., 2017; Parkes et al., 2018) was less germane. Instead, the anatomical CompCor approach of noise reduction was used (Behzadi et al., 2007). Anatomical volumes were segmented into grey matter, white matter, and cerebrospinal fluid areas, and the resulting white matter and cerebrospinal fluid masks were eroded (one voxel erosion) to minimize partial volume effects. Nuisance variables were modeled as temporal covariates and removed from the blood oxygen level–dependent (BOLD) time series functional data using linear regression. These covariates included estimated subject motion (3 rotation and 3 translation parameters, plus another 6 parameters representing their first-order temporal derivatives) and the BOLD time series data outside the subject-specific grey matter mask (i.e., 3 temporal PCA components from the subject-specific white matter mask and 3 temporal PCA parameters from the cerebrospinal fluid mask). The resulting BOLD time series was band-pass filtered (0.008 Hz < f < 0.08 Hz).

### DMN seed definition

The seed ROI was defined to reflect the DMN and the abnormally high functionally connected subgenual prefrontal cortex reported in individuals with MDD (Hamilton et al., 2015). Two commonly used DMN nodes, the posterior cingulate cortex (−5, −49, 40) and medial prefrontal cortex (−1, 47, −4; Fox et al., 2005), were combined with the subgenual prefrontal cortex (0, 26, −10) as a seed region. The WFU PickAtlas (Maldjian et al., 2004; Maldjian et al., 2003) was used to create 6-mm radius ROIs in MNI space for each of these three regions.

### Subject-level seed to voxel analyses

Subject-level seed to voxel FC maps were generated with the preprocessed BOLD time course data. The three DMN-subgenual ROIs FC were averaged and then correlated with voxels throughout the remainder of the brain. Whereas our hypothesis based on rTMS therapeutic effects was that the ROI would correlate with a frontal subregion, a whole brain analysis enabled more conservative corrections in case alternative FC was detected (Beynel et al., 2020). This analysis produced subject-level correlation maps that were converted to z scores using Fisher’s r-to-z transformation.

### Group seed to voxel analyses

Group-based analyses were completed by correlating subject-level maps with reflection or brooding. Additionally, while our interests are primarily in the reflection and brooding subscales, to enable comparison to the previous literature we also evaluated the RRS total score, correlating the RRS total score with subject-level maps. Cluster thresholds were defined with a *p*_uncorrected_ < .001 and corrected for multiple comparisons with a two-sided *p*_FDR_ < .05. These analyses were repeated with the same statistical threshold while controlling for variables of no interest including demographic (age and sex) and clinical variables (PCL-M and BDI-II). Analyses with the addition of covariates provided additional information that the relationships between brain regions and rumination subtypes were selective and not better accounted for by depression severity or other comorbid and otherwise confounding variables. All seed to voxel analyses controlled for the effect of fMRI scanner site with four, individual by site, dummy coded variables (i.e., each variable contained 1 s for subjects scanned at a site and 0 s for subjects from other sites, repeated for four of five sites; these variables were controlled for in the general linear model). Additionally, to demonstrate that fMRI scanner site did not significantly alter results, significant results were analyzed without the fMRI scanner dummy coded variables while employing the same cluster threshold (*p*_uncorrected_ < .001) and multiple comparison controls (two-sided *p*_FDR_ < .05).

## Results

### Patient characteristics

Table 1 shows descriptive statistics for the demographic and questionnaire data. On average, participants reported moderate depression severity ($\overline{x}$ = 23.3; SD = 10.26) with approximately 60% of the participants experiencing depression severity in the moderate to severe range at the time of fMRI data acquisition. Seven participants received active rTMS treatment on average 21.07 (SD = 9.34) months prior to their fMRI data acquisition. To confirm our findings were not explained by these participants, significant seed to voxel analyses were repeated while controlling for the effect of these seven post-active rTMS participants (i.e., an additional dummy coded variable for these seven participants). The number of participants per fMRI scanner site are found in Supplementary Material Table 1 and correlations between the questionnaire results are found in Supplementary Material Table 2.

### Regions correlated with DMN-subgenual and rumination subtypes and total score

**Fig. 1:**
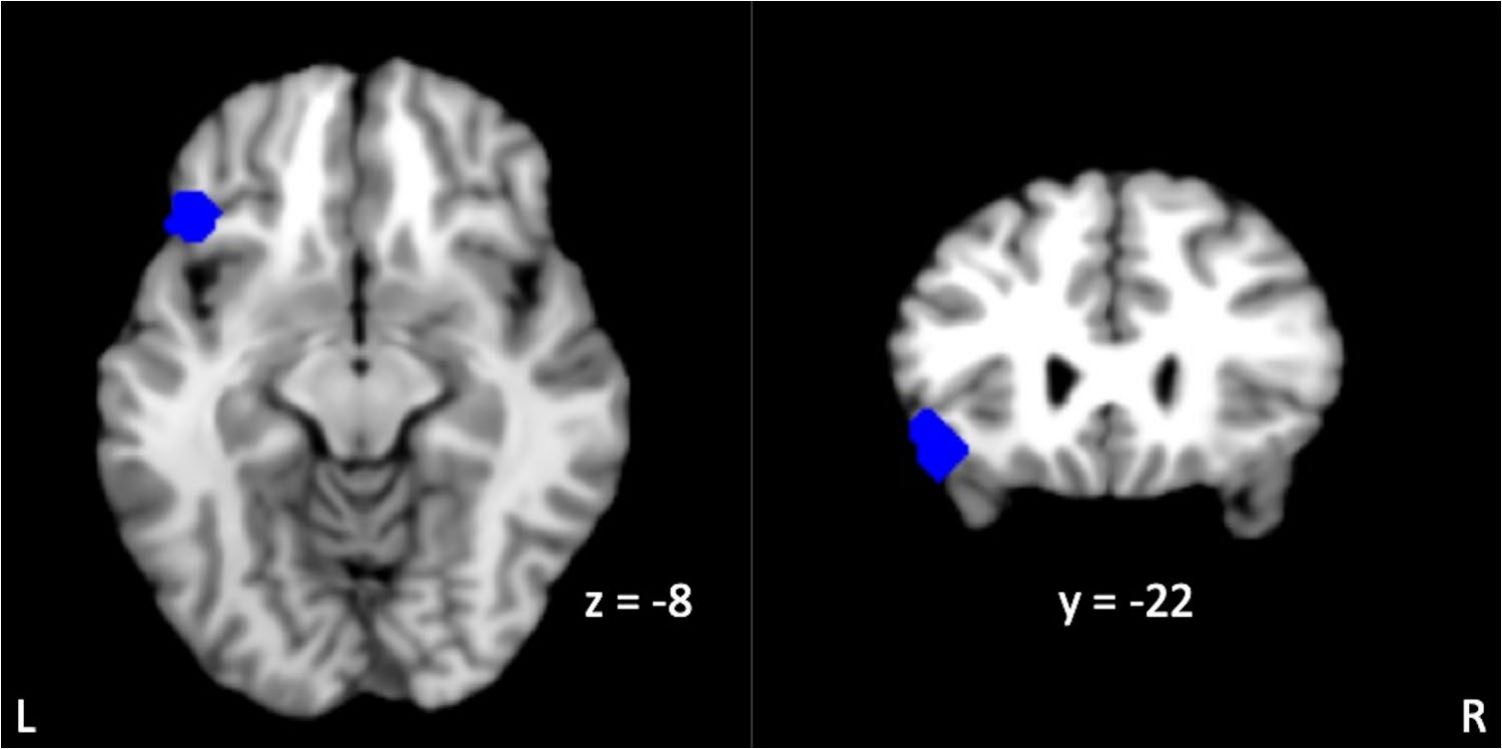
Subject-level ROI-ROI analyses results. Significant seed to voxel analyses (*p*_uncorrected_ < .001 and two-sided *p*_FDR_ < .05) in which higher reflection scores are associated with a stronger anticorrelation between the seeded DMN-subgenual with the left lateral orbitofrontal cortex

**Fig. 2:**
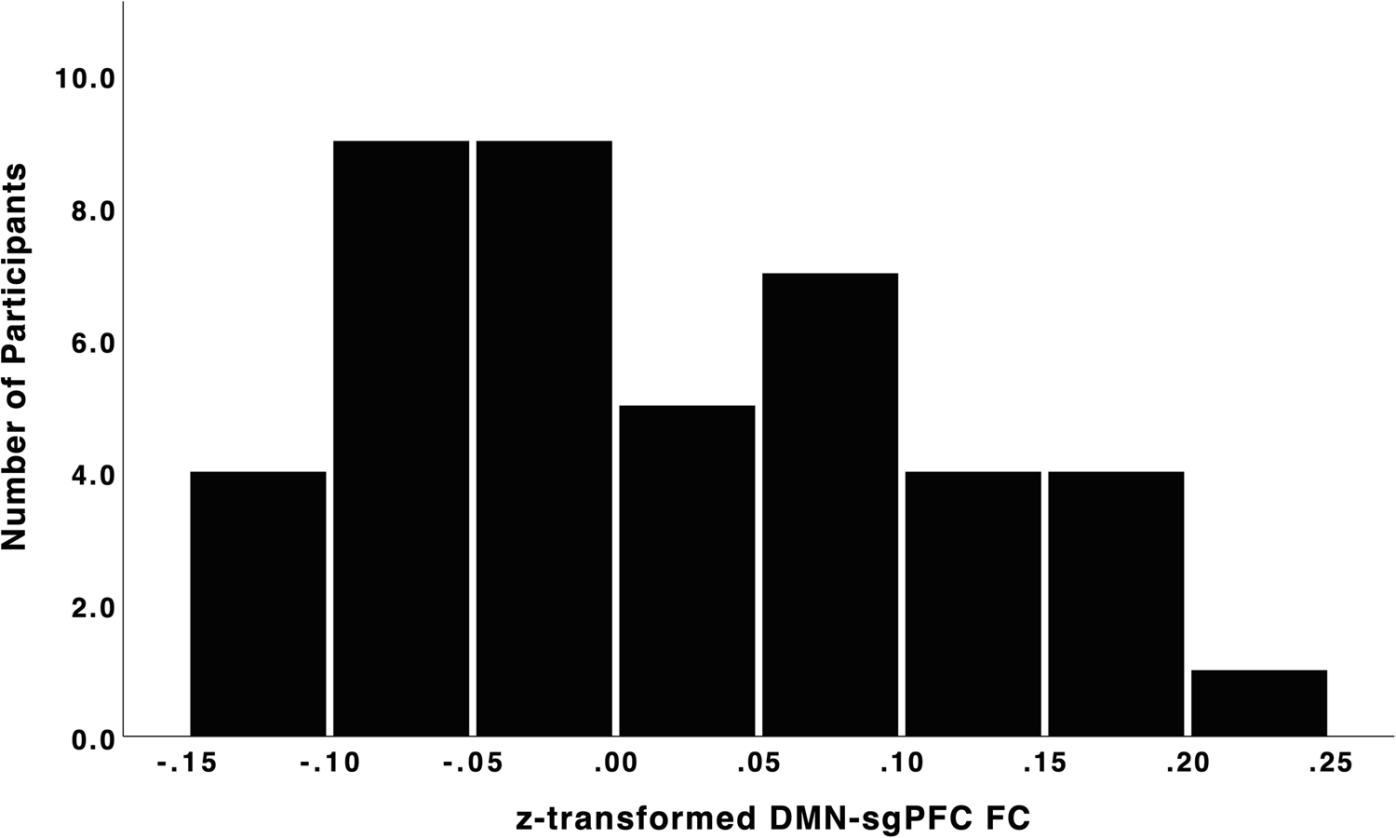
Subject-level FC between the DMN-subgenual and the left orbitofrontal cortex. Subject-level FC between the DMN-subgenual and the left orbitofrontal cortex. The left lateral orbitofrontal cortex was identified with the seed-voxel analyses of the relationship between reflection and the DMN-subgenual seed. These z-transformed values have a group mean of .014 and standard deviation of .095, demonstrating that there is, on average, positive FC between the DMN-subgenual with the left lateral orbitofrontal cortex

**Fig. 3:**
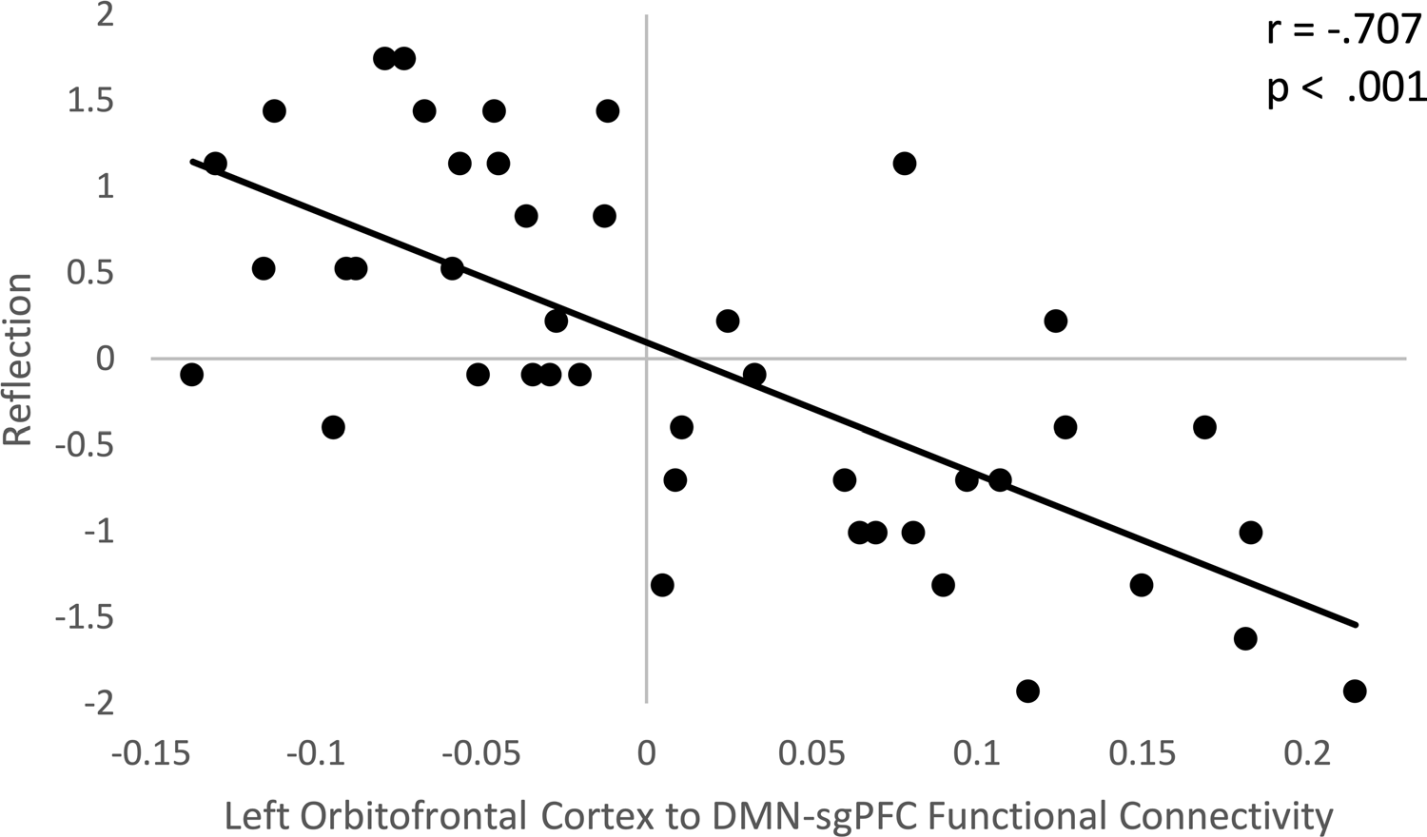
Scatterplot of correlation between reflection with FC (cluster means) between the DMN-subgenual and the left lateral orbitofrontal cortex. Scatterplot depicting the Pearson correlation between the z-transformed reflection subscale of the Rumination Response Scale and the strength of FC (cluster means) between the DMN-subgenual seed with the left lateral orbitofrontal cortex. The FC values were derived from DMN-subgenual seed to voxel analysis with *p*_uncorrected_ < .001 and two-sided *p*_FDR_ < .05

Higher reflection scores were associated (two-sided *p*_FDR_ < .05) with FC between a subregion within the left lateral orbitofrontal cortex (peak −44, 26, −8; *k* = 172) and the DMN-subgenual seed. Seed to voxel analyses comparing brooding and the RRS total score with FC between the DMN-subgenual and voxels in the remainder of the brain were not significant using the same statistical threshold (two-sided *p*_FDR_ > .05), hence no further analyses associated with brooding or the RRS total score are reported. Figure 1 depicts the spatial extent of the reflection-related cluster in the frontal lobe that extended beyond the left lateral orbitofrontal cortex peak to include the inferior frontal gyrus-pars triangularis, frontal operculum, and inferior frontal gyrus-pars opercularis. Individual participant FC values between the DMN-subgenual seed with the left lateral orbitofrontal cortex are shown in Fig. 2, which average to a positive correlation between these regions. Correlations between reflection and FC between the left lateral orbitofrontal cortex and the DMN-subgenual are represented in Fig. 3. This figure is displayed for descriptive purposes (Vul & Pashler, 2012). Overall, there is a negative correlation between the self-reported mental process of reflection and FC between the left lateral orbitofrontal with the DMN-subgenual. Further details are displayed in Table 2. The addition of demographic and clinical variable to the analyses continued to produce significant results (Table 2). Seed to voxel analyses with the addition of a control variable for the effect of the 7 participants with active rTMS prior to fMRI data acquisition produced similar results (Supplemental Material Table 3). Removal of the fMRI scanner site dummy coded variables from the significant seed to voxel analyses produced similar results, except for one analysis in which an additional right frontal pole cluster was significant (Supplemental Material Tables 4 and 5). The additional right frontal pole cluster (peak −46, 26, −8; *k* = 96) was identified in the seed to voxel analysis that evaluated the relationship between reflection while controlling for demographic and clinical variable.

**Table 2:** Significant seed to voxel results

Label	Brodmann Area	Peak Coordinates			Two-sided *p*_FDR_	*t*	Number of voxels (*k*)	Mean (SD)
		x	y	z				
Reflection								
Left lateral orbitofrontal cortex	47	−44	26	−8	.001	−6.25	172	.014 (.095)
Cluster subregions: orbitofrontal cortex = 107 voxels; inferior frontal gyrus pars triangularis = 28 voxels; frontal operculum = 11 voxels; inferior frontal gyrus pars opercularis = 2; not-labeled = 24 voxels								
Reflection w/ Covariates								
Left lateral orbitofrontal cortex	47	−50	26	−10	.014	−5.37	108	.016 (.099)
Cluster subregions: orbitofrontal cortex = 52 voxels; inferior frontal gyrus pars triangularis = 35 voxels; frontal operculum = 4 voxels; not-labeled = 17 voxels								

All analyses controlled for the effects of fMRI scanner site. Covariates = age, sex, posttraumatic stress severity (PCL-M), and depression severity (BDI-II). Brodmann Area = area associated with peak MNI coordinates; *t* = 37 dof for primary analyses and 33 dof for analyses with covariates; Mean (SD) are Fisher r-to-z transformed functional connectivity values

## Discussion

In this study, results indicate that increased reflection was associated with a stronger anticorrelation between a focal region with in the left lateral orbitofrontal cortex and the DMN-subgenual area, but no significant relationships were found between brooding or the RRS total score with FC between the DMN-subgenual and the remainder of the brain. These results extend the literature relating symptoms of treatment resistant depression and FC. Notably, the association between FC and reflection suggests that modulating this network of regions may alter a potentially compensatory process rather than a process that is destructive such as brooding.

These results are consistent with previous rTMS studies suggesting depression can be treated through directly stimulating orbital frontal regions and those frontal regions with anticorrelations with the subgenual region. Whereas modulating dorsolateral prefrontal cortical regions anticorrelated with the subgenual has been associated with more effective rTMS response (Cash et al., 2017; Fox et al., 2012; Mir-Moghtadaei et al., 2015; Weigand et al., 2018; Williams et al., 2018), other frontal regions have been suggested as rTMS targets such as a lateral orbitofrontal cortex (Downar & Daskalakis, 2013). Stimulation of the right orbitofrontal region in patients undergoing rTMS (Feffer et al., 2018) and invasive brain stimulation to left or right orbitofrontal regions (Rao et al., 2018) have been shown to decrease depression severity. Our findings demonstrating the longitudinally adaptive process of reflection is associated an anticorrelation between the orbitofrontal and the DMN-subgenual is consistent with other studies that have demonstrated that an increased anticorrelation between the orbitofrontal region and the DMN is adaptive for depression (Cheng et al., 2018; Jacobs et al., 2016), although our findings are in the left and the other findings are in the right hemisphere. Together, all three studies support that strengthening the anticorrelation between the DMN with the orbitofrontal cortex is associated with positive prognostic factors.

Our findings of an anticorrelation between the orbitofrontal cortex and the DMN-subgenual region is of particular interest because the orbitofrontal cortex is one of two prefrontal regions associated with rumination in major depressive disorder (Cooney et al., 2010). Specifically, during a rumination induction task, individuals diagnosed with major depressive disorder have greater activity than controls in the subgenual cingulate, orbitofrontal cortex, and dorsolateral prefrontal cortex (Cooney et al., 2010). While there is abundant evidence that targeting the dorsolateral prefrontal cortex with rTMS is effective for treating depression (Cash et al., 2017; Fox et al., 2012; Mir-Moghtadaei et al., 2015; Philip et al., 2018; Weigand et al., 2018; Williams et al., 2018), the orbitofrontal cortex is also demonstrating promise as a rTMS target for depression (Feffer et al., 2018). Our findings lend additional evidence that the orbitofrontal cortex may prove to be an effective target with rTMS for depression.

There are limitations to this study. Our participants were exclusively Veterans, many of whom were comorbid for posttraumatic stress disorder (Yesavage et al., 2018). Whereas clinician administered measures were used for the original diagnosis of posttraumatic stress disorder and major depressive disorder, symptom severity of posttraumatic stress disorder (PCL-M) and depression (BDI-II) were also collected at the time of MRI data acquisition in case there was change from the time of diagnosis. Whereas self-report measures are less burdensome and robust against variations in clinical rater biases, self-report measures may have been vulnerable to patient under or over-reporting. The relationship between reflection and DMN-subgenual FC was preserved while controlling for posttraumatic stress disorder symptom severity and other demographic and clinical variables; however, replication in other cohorts is needed. Our participants had a range from positive to negative FC between the orbitofrontal and the DMN-subgenual. While stimulation of the orbitofrontal region has been shown to be effective in decreasing depression severity (Feffer et al., 2018; Rao et al., 2018), it is not known if the strength of FC between the orbitofrontal and the DMN-subgenual is an individual difference that will relate to treatment response. This further highlights the need for a trial of rTMS to the left orbitofrontal cortex. These results are also correlational and cross-sectional, therefore, neuromodulation studies that include rumination as an outcome measure are needed. Furthermore, evaluating relationships between reflection and DMN-subgenual FC in a control population would help to strengthen the specificity of the present findings.

This study addressed a focused question about the nature of rumination and a focal abnormality in highly treatment resistant depressed patients (Hamilton et al., 2015); however, the study of perseverative cognition is a rich and growing literature (for a review see Makovac et al., 2020). Though resting-state fMRI is increasingly used to identify rTMS targets, future work should investigate the dynamics of networks (Chang & Glover, 2010) and further fMRI task-defined and resting-state regions identified by brooding (Kaiser et al., 2018; Vanderhasselt et al., 2013; Vanderhasselt et al., 2011) could be targeted with rTMS.

## Conclusions

We demonstrated that increased reflection and not brooding or the RRS total-score is associated with an anticorrelation between the left lateral orbitofrontal region and the rumination-associated DMN-subgenual network of regions. This association suggests that modulating this system would alter a process believed to be compensatory.

## Supplementary Information

ESM 1 (DOCX 27 kb)

## References

[CR1] Baxter, L. R., Jr., Schwartz, J. M., Phelps, M. E., Mazziotta, J. C., Guze, B. H., Selin, C. E., ..., Sumida, R. M. (1989). Reduction of prefrontal cortex glucose metabolism common to three types of depression. *Arch Gen Psychiatry*, *46*(3), 243–250. 10.1001/archpsyc.1989.01810030049007. 10.1001/archpsyc.1989.01810030049007 2784046

[CR2] Beck AT, Steer RA, Brown GK (1996). Beck depression inventory: Second edition manual.

[CR3] Behzadi Y, Restom K, Liau J, Liu TT (2007). A component based noise correction method (CompCor) for BOLD and perfusion based fMRI. Neuroimage.

[CR4] Berlim MT, McGirr A, Van den Eynde F, Fleck MP, Giacobbe P (2014). Effectiveness and acceptability of deep brain stimulation (DBS) of the subgenual cingulate cortex for treatment-resistant depression: A systematic review and exploratory meta-analysis. Journal of Affective Disorders.

[CR5] Beynel, L., Powers, J. P., & Appelbaum, L. G. (2020). Effects of repetitive transcranial magnetic stimulation on resting-state connectivity: A systematic review. *Neuroimage*, *211*, Article 116596. 10.1016/j.neuroimage.2020.116596. 10.1016/j.neuroimage.2020.116596 PMC7571509 32014552

[CR6] Blake, D., Weathers, F., Nagy, L., Kaloupek, D., & Gusman, F. C., Charney, DS, & Keane, TM (1995). *Clinician-Administered PTSD Scale for DSM-IV (CAPS-DX)*. 10.1007/BF02105408 7712061

[CR7] Brown, G. G., Mathalon, D. H., Stern, H., Ford, J., Mueller, B., Greve, D. N., ..., Potkin, S. G. (2011). Multisite reliability of cognitive BOLD data. *Neuroimage*, *54*(3), 2163–2175. 10.1016/j.neuroimage.2010.09.076. 10.1016/j.neuroimage.2010.09.076 PMC3009557 20932915

[CR8] Cash, R. F. H., Noda, Y., Zomorrodi, R., Radhu, N., Farzan, F., Rajji, T. K., ..., Blumberger, D. M. (2017). Characterization of glutamatergic and GABA(a)-mediated neurotransmission in motor and dorsolateral prefrontal cortex using paired-pulse TMS-EEG. *Neuropsychopharmacology*, *42*(2), 502–511. 10.1038/npp.2016.133. 10.1038/npp.2016.133 PMC5399228 27461082

[CR9] Chai XJ, Castanon AN, Ongur D, Whitfield-Gabrieli S (2012). Anticorrelations in resting state networks without global signal regression. Neuroimage.

[CR10] Chang C, Glover GH (2010). Time-frequency dynamics of resting-state brain connectivity measured with fMRI. Neuroimage.

[CR11] Cheng, W., Rolls, E. T., Qiu, J., Xie, X., Wei, D., Huang, C. C., ..., Feng, J. (2018). Increased functional connectivity of the posterior cingulate cortex with the lateral orbitofrontal cortex in depression. *Transl Psychiatry*, *8*(1), 90. 10.1038/s41398-018-0139-1. 10.1038/s41398-018-0139-1 PMC5915597 29691380

[CR12] Ciric, R., Wolf, D. H., Power, J. D., Roalf, D. R., Baum, G. L., Ruparel, K., ..., Davatzikos, C. (2017). Benchmarking of participant-level confound regression strategies for the control of motion artifact in studies of functional connectivity. *Neuroimage*, *154*, 174–187. 10.1016/j.neuroimage.2017.03.020 PMC5483393 28302591

[CR13] Cooney RE, Joormann J, Eugène F, Dennis EL, Gotlib IH (2010). Neural correlates of rumination in depression. Cognitive, Affective, & Behavioral Neuroscience.

[CR14] Downar J, Daskalakis ZJ (2013). New targets for rTMS in depression: A review of convergent evidence. Brain Stimulation.

[CR15] Drobisz D, Damborska A (2019). Deep brain stimulation targets for treating depression. Behavioural Brain Research.

[CR16] Feffer K, Fettes P, Giacobbe P, Daskalakis ZJ, Blumberger DM, Downar J (2018). 1Hz rTMS of the right orbitofrontal cortex for major depression: Safety, tolerability and clinical outcomes. European Neuropsychopharmacology.

[CR17] Fox, M. D., Snyder, A. Z., Vincent, J. L., Corbetta, M., Van Essen, D. C., & Raichle, M. E. (2005). The human brain is intrinsically organized into dynamic, anticorrelted functional networks. *Proc Natl Acad Sci U S a*, *102*(27), 9673-9678. 10/1073/pnas.0504136102. 10.1073/pnas.0504136102 PMC1157105 15976020

[CR18] Fox MD, Buckner RL, White MP, Greicius MD, Pascual-Leone A (2012). Efficacy of transcranial magnetic stimulation targets for depression is related to intrinsic functional connectivity with the subgenual cingulate. Biological Psychiatry.

[CR19] Fox MD, Buckner RL, Liu HS, Chakravarty MM, Lozano AM, Pascual-Leone A (2014). Resting-state networks link invasive and noninvasive brain stimulation across diverse psychiatric and neurological diseases [article]. Proceedings of the National Academy of Sciences of the United States of America.

[CR20] George MS, Wassermann EM (1994). Rapid-rate transcranial magnetic stimulation and ECT. Convulsive Therapy.

[CR21] Greve, D. N., Mueller, B. A., Liu, T., Turner, J. A., Voyvodic, J., Yetter, E., ..., Glover, G. (2011). A novel method for quantifying scanner instability in fMRI. *Magn Reson. Med*, *65*(4), 1053–1061. 10.1002/mrm.22691. 10.1002/mrm.22691 PMC3117086 21413069

[CR22] Hamilton M (1960). A rating scale for depression. Journal of Neurology, Neurosurgery, and Psychiatry.

[CR23] Hamilton JP, Farmer M, Fogelman P, Gotlib IH (2015). Depressive rumination, the default-mode network, and the dark matter of clinical neuroscience. Biological Psychiatry.

[CR24] Jacobs RH, Watkins ER, Peters AT, Feldhaus CG, Barba A, Carbray J, Langenecker SA (2016). Targeting ruminative thinking in adolescents at risk for depressive relapse: Rumination-focused cognitive behavior therapy in a pilot randomized controlled trial with resting state fMRI. PLoS One.

[CR25] Kaiser RH, Snyder HR, Franziska G, Clegg R, Ironside M, Pizzagalli DA (2018). Attention bias in rumination and depression: Cognitive mechanisms and brain networks. Clinical Psychological Science.

[CR26] Keator, D. B., van Erp, T. G., Turner, J. A., Glover, G. H., Mueller, B. A., Liu, T. T., ..., Fbirn. (2016). The function biomedical informatics research network data repository. *Neuroimage*, *124*(Pt B), 1074–1079. 10.1016/j.neuroimage.2015.09.003. 10.1016/j.neuroimage.2015.09.003 PMC4651841 26364863

[CR27] Lantrip C, Gunning FM, Flashman L, Roth RM, Holtzheimer PE (2017). Effects of transcranial magnetic stimulation on the cognitive control of emotion: Potential antidepressant mechanisms [review]. Journal of Ect.

[CR28] Makovac E, Fagioli S, Rae CL, Critchley HD, Ottaviani C (2020). Can't get it off my brain: Meta-analysis of neuroimaging studies on perseverative cognition. Psychiatry Research: Neuroimaging.

[CR29] Maldjian JA, Laurienti PJ, Kraft RA, Burdette JH (2003). An automated method for neuroanatomic and cytoarchitectonic atlas-based interrogation of fMRI data sets. Neuroimage.

[CR30] Maldjian JA, Laurienti PJ, Burdette JH (2004). Precentral gyrus discrepancy in electronic versions of the Talairach atlas. Neuroimage.

[CR31] Martinot, M. L. P., Galinowski, A., Ringuenet, D., Gallarda, T., Lefaucheur, J. P., Bellivier, F., ..., Martinot, J. L. (2010). Influence of prefrontal target region on the efficacy of repetitive transcranial magnetic stimulation in patients with medication-resistant depression: a [(18)F]-fluorodeoxyglucose PET and MRI study. *International Journal of Neuropsychopharmacology*, *13*(1), 45–59. 10.1017/S146114570900008X 19267956

[CR32] Mayberg, H. S., Liotti, M., Brannan, S. K., McGinnis, S., Mahurin, R. K., Jerabek, P. A., ..., Lancaster, J. L. (1999). Reciprocal limbic-cortical function and negative mood: Converging PET findings in depression and normal sadness. *American journal of psychiatry*, *156*(5), 675–682. 10.1176/ajp.156.5.675 10327898

[CR33] Mi, Z., Biswas, K., Fairchild, J. K., Davis-Karim, A., Phibbs, C. S., Forman, S. D., ..., Yesavage, J. A. (2017). Repetitive transcranial magnetic stimulation (rTMS) for treatment-resistant major depression (TRMD) veteran patients: Study protocol for a randomized controlled trial. *Trials*, *18*(1), 409. 10.1186/s13063-017-2125-y. 10.1186/s13063-017-2125-y PMC5581925 28865495

[CR34] Mir-Moghtadaei, A., Caballero, R., Fried, P., Fox, M. D., Lee, K., Giacobbe, P., ..., Downar, J. (2015). Concordance between BeamF3 and MRI-neuronavigated target sites for repetitive transcranial magnetic stimulation of the left dorsolateral prefrontal cortex. *Brain Stimul*, *8*(5), 965–973. 10.1016/j.brs.2015.05.008. 10.1016/j.brs.2015.05.008 PMC4833442 26115776

[CR35] Morishita T, Fayad SM, Higuchi MA, Nestor KA, Foote KD (2014). Deep brain stimulation for treatment-resistant depression: Systematic review of clinical outcomes. Neurotherapeutics.

[CR36] Murphy K, Birn RM, Handwerker DA, Jones TB, Bandettini PA (2009). The impact of global signal regression on resting state correlations: Are anti-correlated networks introduced?. Neuroimage.

[CR37] Nolen-Hoeksema S, Morrow J (1991). A prospective study of depression and posttraumatic stress symptoms after a natural disaster: The 1989 Loma Prieta earthquake. Journal of Personality and Social Psychology.

[CR38] Parkes L, Fulcher B, Yücel M, Fornito A (2018). An evaluation of the efficacy, reliability, and sensitivity of motion correction strategies for resting-state functional MRI. Neuroimage.

[CR39] Philip NS, Barredo J, Aiken E, Carpenter LL (2018). Neuroimaging mechanisms of therapeutic transcranial magnetic stimulation for major depressive disorder. Biological Psychiatry: Cognitive Neuroscience and Neuroimaging.

[CR40] Rao, V. R., Sellers, K. K., Wallace, D. L., Lee, M. B., Bijanzadeh, M., Sani, O. G., ..., Chang, E. F. (2018). Direct electrical stimulation of lateral orbitofrontal cortex acutely improves mood in individuals with symptoms of depression. *Curr Biol*, *28*(24), 3893–3902 e3894. 10.1016/j.cub.2018.10.026. 10.1016/j.cub.2018.10.026 30503621

[CR41] Saad ZS, Gotts SJ, Murphy K, Chen G, Jo HJ, Martin A, Cox RW (2012). Trouble at rest: How correlation patterns and group differences become distorted after global signal regression. Brain Connectivity.

[CR42] Treynor W, Gonzalez R, Nolen-Hoeksema S (2003). Rumination reconsidered: A psychometric analysis. Cognitive Therapy and Research.

[CR43] Vanderhasselt MA, Kuhn S, De Raedt R (2011). Healthy brooders employ more attentional resources when disengaging from the negative: An event-related fMRI study. Cognitive, Affective, & Behavioral Neuroscience.

[CR44] Vanderhasselt M-A, Baeken C, Van Schuerbeek P, Luypaert R, De Mey J, De Raedt R (2013). How brooding minds inhibit negative material: An event-related fMRI study. Brain and Cognition.

[CR45] Vul E, Pashler H (2012). Voodoo and circularity errors. Neuroimage.

[CR46] Weathers, F. W., Litz, B. T., Herman, D. S., Huska, J. A., & Keane, T. M. (1993). PTSD checklist-military version. *PsycTESTS Dataset.*10.1037/t05198-000

[CR47] Weigand, A., Horn, A., Caballero, R., Cooke, D., Stern, A. P., Taylor, S. F., ..., Fox, M. D. (2018). Prospective validation that Subgenual connectivity predicts antidepressant efficacy of transcranial magnetic stimulation sites. *Biological Psychiatry*, *84*(1), 28–37. 10.1016/j.biopsych.2017.10.028 PMC6091227 29274805

[CR48] Whitfield-Gabrieli S, Nieto-Castanon A (2012). Conn: A functional connectivity toolbox for correlated and anticorrelated brain networks. Brain Connectivity.

[CR49] Williams, N. R., Sudheimer, K. D., Bentzley, B. S., Pannu, J., Stimpson, K. H., Duvio, D., ..., Schatzberg, A. F. (2018). High-dose spaced theta-burst TMS as a rapid-acting antidepressant in highly refractory depression. *Brain*, *141*(3), e18-e18. 10.1093/brain/awx379. 10.1093/brain/awx379 PMC5837258 29415152

[CR50] Yesavage, J. A., Fairchild, J. K., Mi, Z., Biswas, K., Davis-Karim, A., Phibbs, C. S., ..., Team, V. A. C. S. P. S. (2018). Effect of Repetitive Transcranial Magnetic Stimulation on Treatment-Resistant Major Depression in US Veterans: A Randomized Clinical Trial. *JAMA Psychiatry*, *75*(9), 884–893. 10.1001/jamapsychiatry.2018.1483. 10.1001/jamapsychiatry.2018.1483 PMC6142912 29955803

