# Supplementary material for: Ruminative reflection is associated with anticorrelations between the orbitofrontal cortex and the default mode network in depression: implications for repetitive transcranial magnetic stimulation
Source: Brain Imaging Behav. 2021 Dec 3;16(3):1186–95. doi: 10.1007/s11682-021-00596-4 (PMC9107429; doi:10.1007/s11682-021-00596-4)
Supplement: Supplementary file 1 — (DOCX 27 kb) [file 11682_2021_596_MOESM1_ESM.docx]

**Supplemental Material**

Table 1. Number of subjects per fMRI scanner site.

| Site | N |
| --- | --- |
| Charleston | 6 |
| Palo Alto | 11 |
| Pittsburgh | 12 |
| Salt Lake City | 7 |
| San Francisco | 7 |

Table 2. Correlations of Covariates

|  | Rumination | Reflection | Brooding | BDI-II |
| --- | --- | --- | --- | --- |
| Reflection | .61** |  |  |  |
| Brooding | .83** | .48** |  |  |
| BDI-II | .36* | -.01 | .25 |  |
| PCL-M | .21 | .16 | .19 | .34* |

*Note.* Rumination = total score of the Rumination Response Scale; Reflection = reflection subscale of the Rumination Response Scale; Brooding = brooding subscale of the Rumination Response Scale; BDI-II = Beck Depression Inventory-II; PCL-M = Posttraumatic Stress Disorder Checklist, Military Version.

*p < .05; **p < .001.

Table 3. Significant seed to voxel results controlled for participants with active rTMS prior to fMRI scan

| Label | |  | Brodmann Area | | | | Peak Coordinates  x y z | | | | | Two-sided *p*_FDR_ | *t* | | Number of voxels (*k*) | | | | |  |
| --- | --- | --- | --- | --- | --- | --- | --- | --- | --- | --- | --- | --- | --- | --- | --- | --- | --- | --- | --- | --- |
| Reflection | | |  |  |  |  |  |  |  |  |  |  |  |  |  |  |  |  |  |  |
|  | Left lateral orbitofrontal cortex and inferior frontal gyrus | | | 47 | | -44 | | 26 | | -8 | < .001 | | | -6.36 | | 177 | | |  |  |
| Reflection w/ Covariates | | | | |  | |  |  |  |  | |  | | |  | |  |  |  |  |
|  | Left lateral orbitofrontal cortex and inferior frontal gyrus | | | 47 | | -48 | | 26 | | -6 | .013 | | | -5.33 | | 108 | | | | |

*note.* Results of analyses evaluating reflection while controlling for seven subjects who had their fMRI data collected post-active rTMS. All analyses controlled for the effects of fMRI scanner site. Covariates = age, sex, posttraumatic stress severity (PCL-M), and depression severity (BDI-II), participants with fMRI post-active treatment. Brodmann Area = area associated with peak MNI coordinates; *t* = 36 dof for primary analyses and 32 dof for analyses with covariates; Mean (SD) are Fisher r-to-z transformed functional connectivity values.

Table 4. Significant seed to voxel results without fMRI scanner site control variables.

| Label | |  | Brodmann Area | | | | Peak Coordinates  x y z | | | | | Two-sided *p*_FDR_ | *t* | | Number of voxels (*k*) | | | |  |
| --- | --- | --- | --- | --- | --- | --- | --- | --- | --- | --- | --- | --- | --- | --- | --- | --- | --- | --- | --- |
| Reflection | | |  |  |  |  |  |  |  |  |  |  |  |  |  |  |  |  |  |
|  | Left lateral orbitofrontal cortex | | | 47 | | -44 | | 26 | | -8 | .003 | | | -3.54 | | 161 | |  |  |
| Reflection w/ Covariates | | | | |  | |  |  |  |  | |  | | |  | |  |  |  |
|  | Left lateral orbitofrontal cortex | | | 47 | | -48 | | 26 | | -10 | .015 | | | -3.57 | | 115 | | | |
|  | Right Frontal Pole | | | 10 | | 44 | | 58 | | -12 | .018 | | | -3.57 | | 96 | | | |

*note.* Analyses were not controlled for the effects of fMRI scanner site. Covariates = age, sex, posttraumatic stress severity (PCL-M), and depression severity (BDI-II). Brodmann Area = area associated with peak MNI coordinates; *t* = 41 dof for primary analyses and 37 dof for analyses with covariates; Mean (SD) are Fisher r-to-z transformed functional connectivity values.

Table 5. Significant seed to voxel results controlled for participants with active rTMS prior to fMRI scan without fMRI scanner site control variables

| Label | |  | Brodmann Area | | | | Peak Coordinates  x y z | | | | | Two-sided *p*_FDR_ | *t* | | Number of voxels (*k*) | | | |  |
| --- | --- | --- | --- | --- | --- | --- | --- | --- | --- | --- | --- | --- | --- | --- | --- | --- | --- | --- | --- |
| Reflection | | |  |  |  |  |  |  |  |  |  |  |  |  |  |  |  |  |  |
|  | Left lateral orbitofrontal cortex and inferior frontal gyrus | | | 47 | | -44 | | 26 | | -8 | .001 | | | -3.55 | | 178 | |  |  |
| Reflection w/ Covariates | | | | |  | |  |  |  |  | |  | | |  | |  |  |  |
|  | Left lateral orbitofrontal cortex and inferior frontal gyrus | | | 47 | | -46 | | 26 | | -8 | .020 | | | -3.58 | | 112 | | | |

*note.* Results of analyses evaluating reflection while controlling for seven subjects who had their fMRI data collected post-active rTMS, not controlled for the effects of fMRI scanner site. All analyses controlled for the effects of fMRI scanner site. Covariates = age, sex, posttraumatic stress severity (PCL-M), and depression severity (BDI-II), participants with fMRI post-active treatment. Brodmann Area = area associated with peak MNI coordinates; *t* = 40 dof for primary analyses and 36 dof for analyses with covariates; Mean (SD) are Fisher r-to-z transformed functional connectivity values.
